# COVID-19 AND THE CARE OF ASSISTED LIVING RESIDENTS: THE COVCARES PROGRAM OF RESEARCH

**DOI:** 10.1093/geroni/igad104.1627

**Published:** 2023-12-21

**Authors:** Matthias Hoben, Colleen Maxwell

**Affiliations:** York University, Toronto, Ontario, Canada; University of Waterloo, Waterloo, Ontario, Canada

## Abstract

This presentation describes the goals, design and methods of the COVID-19 and the Care of Assisted living Residents (COVCARES) program of research. Our goals were to address critical knowledge gaps related to how COVID-19 impacted assisted living (AL) residents, family/friend caregivers, and homes, and to compare how outcomes differed between AL and nursing homes (NH). Partnering closely with provincial Alzheimer Societies, family caregiver advocates, and health policy decision makers, we conducted two interconnected studies: The first was a prospective cohort study, surveying family/friend caregivers of AL residents and directors of care of AL homes in the Canadian provinces of Alberta and British Columbia twice (10/2020–04/2021 and 07/2021–09/2021). 673 caregivers and 104 AL homes responded to our first survey. Of those, 386 caregivers and 78 AL homes submitted a second survey. The second was a population-based retrospective cohort study (01/2017–12/2021), using clinical and health administrative data of all AL and NH residents in Alberta. Data included Resident Assessment Instrument (RAI) records, acute care and emergency department admission data, prescription medications, data on COVID-19 infections, and vital statistics of 23,355 AL residents and 41,583 NH residents. Analyzing each of the aforementioned data sets individually, and linking family and facility surveys, as well as resident and facility survey data, we provide unique insights into the impacts of the COVID-19 pandemic on AL settings. The presentations in this symposium will share selected key findings from our program of research.

